# Assessment of UTI Diagnostic Techniques Using the Fuzzy–PROMETHEE Model

**DOI:** 10.3390/diagnostics13223421

**Published:** 2023-11-10

**Authors:** Mariam Abobakr, Berna Uzun, Dilber Uzun Ozsahin, Tamer Sanlidag, Ayse Arikan

**Affiliations:** 1 Department of Medical Microbiology and Clinical Microbiology, Near East University, TRNC Mersin 10, Nicosia 99138, Turkey; 2 Department of Mathematics, Near East University, TRNC Mersin 10, Nicosia 99138, Turkey; 3 Department of Medical Diagnostic Imaging, Collage of Health Sciences, University of Sharjah, Sharjah 27272, United Arab Emirates; dozsahin@sharjah.ac.ae; 4 Research Institute of Medical and Health Sciences, University of Sharjah, Sharjah 27272, United Arab Emirates; 5 Operational Research Center in Healthcare, Near East University, TRNC Mersin 10, Nicosia 99138, Turkey; 6 DESAM Research Institute, Near East University, TRNC Mersin 10, Nicosia 99138, Turkey; tamer.sanlidag@neu.edu.tr; 7 Department of Medical Microbiology and Clinical Microbiology, Kyrenia University, TRNC Mersin 10, Kyrenia 99320, Turkey

**Keywords:** urinary tract infection, efficiency, diagnosis, fuzzy logic, decision making, multi criteria analysis

## Abstract

Accurate diagnosis of urinary tract infections (UTIs) is important as early diagnosis increases treatment rates, reduces the risk of infection and disease spread, and prevents deaths. This study aims to evaluate various parameters of existing and developing techniques for the diagnosis of UTIs, the majority of which are approved by the FDA, and rank them according to their performance levels. The study includes 16 UTI tests, and the fuzzy preference ranking organization method was used to analyze the parameters such as analytical efficiency, result time, specificity, sensitivity, positive predictive value, and negative predictive value. Our findings show that the biosensor test was the most indicative of expected test performance for UTIs, with a net flow of 0.0063. This was followed by real-time microscopy systems, catalase, and combined LE and nitrite, which were ranked second, third, and fourth with net flows of 0.003, 0.0026, and 0.0025, respectively. Sequence-based diagnostics was the least favourable alternative with a net flow of −0.0048. The F–PROMETHEE method can aid decision makers in making decisions on the most suitable UTI tests to support the outcomes of each country or patient based on specific conditions and priorities.

## 1. Introduction

Urinary tract infections (UTIs) are inflammations caused by different types of microbial pathogens in the urinary tract; they constitute the second most frequent infectious disorder and are one of the causes contributing to morbidity and mortality [1,2]. UTIs cause more than 7 million clinic visits as well as more than one million hospitalizations and approximately 150 million deaths annually, with incidence rates varying across age groups and genders [2,3]. Early and accurate diagnosis of UTI is essential to prevent the spread of infection. The Food and Drug Administration (FDA) of the United States recently released many techniques for diagnosing UTIs including various conventional techniques [4,5]. Standard dipstick analyses (nitrite and leukocyte esterase tests) are used in urinalysis. While the nitrite test detects bacteria that reduce nitrate to nitrite, the leukocyte esterase test detects the leukocyte enzyme, the level of which increases in the urine during the disease. The disadvantage of these tests is their weakness in terms of negative predictive values (NPVs). Additionally, pathogens that are Gram-positive including *Staphylococcus* spp. and *Enterococcus* spp. cannot be detected as they do not produce nitrites [6,7]. Quantitative urine culture is the standard procedure for the diagnosis of UTIs, but this procedure is laborious, highly time-consuming, and cannot be performed in resource-underprivileged healthcare setups [2]. While the misdiagnosis of an infection leads to an increased prevalence of microbiota, patients experiencing acute UTI attacks are particularly at risk due to altered antimicrobial resistance, which may result in therapeutic failure [2,8,9]. Therefore, new diagnostic technologies that have the potential to be rapid and accurate. In recent years, powerful rapid diagnostic tools have been developed by diagnostic test manufacturers globally to support clinical microbiology professionals; these rapid techniques are promising as they are practical and rapid tools. Urine flow cytometry, a precise and rapid method of counting bacteria in urine specimens, can be used to detect UTIs and may result in a reduction in the need for urine culture, labour, and costs [10,11]. Polymerase chain reaction (PCR) provides a time-saving, cost-effective, rapid method for pathogen identification [6]. However, current PCR techniques only provide qualitative information about the presence of microorganisms, not their concentration [7]. Cell division and bacterial growth in urine can be directly monitored with real-time microscopic imaging. Thus, the results are faster than standard methods [7,12]. Emerging diagnostic platforms such as biosensors and microfluidics are fast point-of-care (POC) tests. A biosensor is a tool that converts biorecognition into a measurable signal of a specific uropathogen. Biosensors (electrochemical, microbial, enzymatic, and optical) have high efficiency but also have a significant cost compared to traditional methods [8,13]. Microfluidic devices are small, effective, and potential diagnostic platforms that can be applied in practice for the detection of harmful microorganisms [14,15]. Pathogenic bacteria in pneumatic microchannels can be identified based on their size and form. The benefits of microfluidic devices are numerous, including lower cost and simplicity, and they can also operate without pumps or additional support tools [16]. Paper-based microfluidic devices are frequently used as diagnostic tools for POC, which can reduce the time and cost compared to laboratory-based analytical techniques. Another new technology that has been developed is called Matrix Assisted Laser Desorption Ionization Time-of-Flight Mass Spectrometry (MALDI–TOF–MS). It is an innovative, accurate and robust approach that has recently been introduced and has translated into new applications of pathogen identification for clinical diagnostic microbiology; this technique has saved clinicians significant time in initiating appropriate treatment for patients with acute UTI episodes [14]. Fluorescence in situ hybridization (FISH), one of the molecular methods, enables fluorescently labelled nucleic acids to bind to the target sequences of the microorganism and create complementary base pairing. The hybrid complex can then be clearly visualized using a fluorescent microscope. Immunology-based tests are rapid, inexpensive tests that are particularly sensitive to Gram-negative bacteria and can detect bacteriuria, but their use is limited due to poor sensitivity against Gram-positive bacteria [6,7,8,9,10,11,12,13,14,15,16,17,18,19,20,21].

In particular, the more options there are, the more difficult the decision becomes, and the complexity increases due to the multitude of methods that can influence the diagnosis. To determine the appropriate tests for UTI diagnosis, support is needed for analytical approaches. Multi-criteria decision-making (MCDM) methods were first developed in the 1960s, the aim of which was to keep the process of decision making (DM) under control and obtain a decision result as fast as possible when there are many options and criteria [22]. There are many MCDM techniques that can aid decision makers in finding the most appropriate solution and ranking the alternative options, such as the Analytic Hierarchy Process (AHP), Technique for Order of Preference by Similarity to Ideal Solution (TOPSIS), Weighted Sum Model (WSM), ELECTRE (Elimination and Choice Expressing Reality), and PROMETHEE (Preference Ranking Organization Method for Enrichment Evaluations) [23]. Each technique has its own strengths and weaknesses, making them suitable for different decision-making contexts and criteria. The choice of the most suitable MCDM technique depends on the specific characteristics of the decision problem and the preferences of the decision maker. However, different approaches can also be used to determine the effectiveness of the decision-making units such as data envelopment analysis (DEA), goal programming, and Grey Relational Analysis (GRA), among others. Abdullah et al. applied MCDA approaches to determine the essential criteria that hold relevance and significance for enhancing flood and drought event management [23]. Wang et al. applied DEA Malmquist and Epsilon-based measure (EBM) approaches to provide an assessment of the efficiency of lithium-ion battery manufacturers after a comprehensive evaluation [24]. Wang et al. discussed MCDM approaches in detail as assistive models for addressing the selection problem of industrial machines. Additionally, they proposed dual MCDM approaches [25]. Mustapha et al. applied MCDA approaches to determine the most effective artificial intelligence (AI)-based models for breast cancer diagnosis prediction [26].

The PROMETHEE method, one of the most recently developed important techniques, was introduced in 1982 by Jean-Pierre Brans and improved in 1985 by Jean-Pierre Brans and Philippe Vincke [27]. The PROMETHEE method is a clear, user-friendly, and balanced method that can manage data when there are multiple parameters [27]. Fuzzy logic can support experts when they are faced with a vague environment, as presented by Zadeh in 1965 [28]. The hybrid model F–PROMETHEE is a beneficial and successfully applied approach for decision makers when the decision matrix contains vague or linguistic information [26].

Recently, various tests have been widely used for the diagnosis of UTIs. In the current study, we aimed to apply the fuzzy PROMETHEE in the evaluation of traditional and newly developed techniques for the diagnosis of UTI and to evaluate and rank them according to their performance by considering their characteristics. The strengths and weaknesses of the tests were also analyzed using this approach.

This study contains five sections. Section 1 presents detailed information about UTIs and the study’s objectives, along with the proposed methodology. Section 2 covers the materials and methodology. In Section 3, the results are provided, and Section 4 covers the discussions. The conclusions are presented in Section 5.

Overall, our study presents the following contributions:

- Discussing detailed features of UTI tests, determining their advantages and disadvantages, and evaluating them analytically for potential enhancements of decision points;
- Guiding the decision makers in deciding the most appropriate and accurate UTI test;
- Applicability of current mathematical approaches in evaluating the test performances used in the diagnosis of infectious diseases.

## 2. Materials and Methods

**Figure 1:**
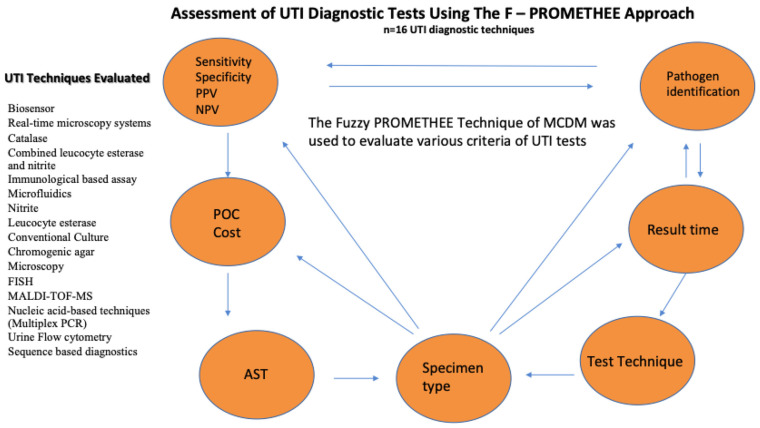
The workflow of the study. **Abbreviations:** POC: point of care; AST: antimicrobial susceptibility testing; MCDM: multi-criteria decision making; FISH; Fluorescence in situ hybridization, MALDI–TOF–MS; matrix-assisted laser desorption ionization-time of flight, PCR; polymerase chain reaction.

The current study includes 16 different UTI testing techniques, including commonly used techniques and emerging technologies in the diagnosis of UTI. Most of the tests have FDA and European Association of Urology (EAU) approval for the diagnosis of UTIs [4,29,30]. Conventional techniques including leukocyte esterase, nitrite, catalase, and culture, as well as developing techniques including FISH, biosensors, microfluidics, nucleic acid-based techniques, immunological-based techniques, sequence-based diagnostics, MALDI–TOF–MS and real-time microscopy systems, were involved in the analysis. Many criteria of test techniques including specimen volume, specimen type, cost, efficiency, result time, point of care testing, applicability/direct from sample, practicality for patients, practicality for personnel, limitation/pathogen identification, sensitivity, specificity, positive predictive value (PPV), NPV, and antimicrobial susceptibility testing (AST) were considered for the evaluation of the UTI testing techniques. The workflow of the study is provided in Figure 1.

**Figure 2:**
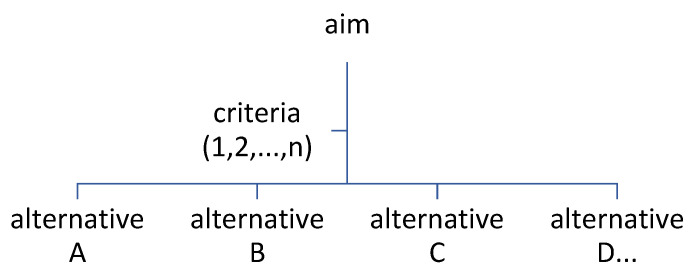
The MCDA main components.

In the study, the MCDM approach was applied using the F–PROMETHEE technique. Ethical approval was not needed, as the study did not involve a human population. The general figure for the MCDA component is provided in Figure 2.

F–PROMETHEE is an MCDM method utilized to clarify the ambiguity parameters of choice problems and compare alternative pairs’ choices for each criterion. One of the most widely utilized methods for making decisions in various fields [26], the F–PROMETHEE approach has recently been applied by numerous researchers in the medical and health fields [26,31,32]. This technique combines the PROMETHEE method with fuzzy logic and aims to simplify the issue into a manageable form, which enables decision makers to incorporate non-crisp parameters into the system, examine them, and rank alternatives based on their criteria [32]. A linguistic scale is used to determine the criterion, which contains vague data; the weight of each criterion is also chosen using a linguistic scale. PROMETHEE I and PROMETHEE II can be, respectively, used to rank the options partially and completely. By assessing the variations in how the choices are ranked, the PROMETHEE method has the ability to rank the alternatives in a system, in order from greatest to least effective. In order to make this assessment, two types of information are needed: the criteria’s weights (relative relevance) and the preference function connected to each criterion and its corresponding alternatives [33]. The preference function $\left(P_{j}\right)$ measures the preference degree of one alternative ($a_{t}$) over another ($a_{t^{\prime }})$.

The Gaussian preference function [27] was selected in this study as provided in Equation (1) since it assigns the preference to the alternatives in a degree, where ‘*d*’ stands for the difference between the options’ considered parameters, and ‘*s*’ is the standard deviation of the selected parameters:

(1) Pd=0, d≤01−e−d22s2x>0

The preference degree ranges from 0 to 1. There are various sorts of preference functions, including the U-shaped function, the usual function, the V-shape function, the level function, the Gaussian function, and the linear function [27]. The PROMETHEE analysis involves the collection of the criteria of the alternatives, the determination of a certain preference function indicated as $P_{j}\left(d\right)$, where each criterion is denoted as *j*, and the weight of the criterion is denoted as a vector $w_{T}=\left(w_{1},\;w_{2},\ldots ,w_{k}\right)$ where j∈(1,…k). The weights can be equal if the criterion’s relative importance is equal [26,27].

The preference indices of $a_{t}$ over $a_{t^{\prime }}$ ∈A has been determined by $\pi \left(a_{t},a_{t^{\prime }}\right)$ and should be computed by applying Equation (2) below.

(2) $$\pi \left(a_{t},a_{t^{\prime }}\right)=\sum_{k=1}^{K}w_{k}\cdot \left[p_{k}\left(f_{k}\left(a_{t}\right)-f_{k}\left(a_{t^{\prime }}\right)\right)\right],\;AXA\to \left[0,1\right]\;$$

π (a,b) represents the preference index, which measures the degree of preference in the multi-criteria decision-making process and k stands for the criteria that were chosen.

The calculation of the positive (or leaving) outranking flow ${(\Phi }^{+}\left(a_{t}\right))$ and negative (or entering) outranking flow ($\Phi^{-}\left(a_{t}\right))$ for the options is as follows in Equations (3) and (4).

(3) $$\Phi^{+}\left(a_{t}\right)=\frac{1}{n-1}\sum_{\underset{t^{\prime }\neq t}{t^{\prime }=1}}^{n}\pi \left(a_{t},a_{t^{\prime }}\right)$$

(4) $$\Phi^{-}\left(a_{t}\right)=\frac{1}{n-1}\sum_{\underset{t^{\prime }\neq t}{t^{\prime }=1}}^{n}\pi \left({a_{t^{\prime }},a}_{t}\right)$$

The number of alternatives is denoted as *n*, where each alternative and the (*n* − 1) number of other options that are available in the system are compared. The leaving flow represented as $\Phi^{+}\left(a_{t}\right)$ demonstrates how strong an alternative is in $a_{t}\in A$, whereas the entering flow denoted as $\Phi^{-}\left(a_{t}\right)$ indicates the weakness of $a_{t}\in A.$ The positive and negative outranking flows are used to calculate the strengths and the weaknesses of the alternatives. The positive outranking flow is the value of one alternative’s support compared to other alternatives based on each criterion. On the other hand, the negative outranking flow denotes the weakness of the alternative compared to other alternatives based on each criterion. The net flow is computed, which is the difference between each alternative’s positive and negative outranking. After the determination of the outranking flows, the partial ranking of the alternatives is assessed by PROMETHEE I, and the net ranking is assessed by PROMETHEE II based on the net flow [27].

In the calculation of the partial order of the alternatives, the alternative that has the highest positive and the lowest negative outranking flows is preferred more than the other alternatives. In PROMETHEE I, alternative $a_{t}$ is preferred over alternative $a_{t^{\prime }}$ ($a_{t}Pa_{t^{\prime }})$ if it satisfies one of the requirements mentioned below as shown in statements in (5).

(5) $$\left\{\begin{array}{c} \Phi^{+}\left(a_{t}\right)>\Phi^{+}\left(a_{t^{\prime }}\right)\;and\;\Phi^{-}\left(a_{t}\right)\leq \Phi^{-}\left(a_{t^{\prime }}\right)\\ \Phi^{+}\left(a_{t}\right)=\Phi^{+}\left(a_{t^{\prime }}\right)\;and\;\Phi^{-}\left(a_{t}\right){<\Phi }^{-}(a_{t^{\prime }}) \end{array}\right.$$

When the alternative $a_{t}$ and the alternative $a_{t^{\prime }}$ have similar leaving and entering flows, $a_{t}$ is indifferent to $a_{t^{\prime }}$ ($a_{t}Ia_{t^{\prime }})$ as shown in a statement in (6):

(6) $${(a}_{t}Ia_{t^{\prime }})\;\mathrm{if}:\;\Phi^{+}\left(a_{t}\right)=\Phi^{+}\left(a_{t^{\prime }}\right)\;and\;\Phi^{-}\left(a_{t}\right){=\Phi }^{-}(a_{t^{\prime }})$$

It is difficult to compare the alternatives with function when a certain alternative has both higher leaving and higher entering outranking flows or lower positive and lower negative outranking flows. Such situations are incomparable with PROMETHEE I [28].

$a_{t}$ is incomparable to alternative $a_{t^{\prime }}$ ${(a}_{t}Ra_{t^{\prime }}$) if it satisfies one of the requirements mentioned in statement (7) below.

(7) $$\left\{\begin{array}{c} \Phi^{+}\left(a_{t}\right)>\Phi^{+}\left(a_{t^{\prime }}\right)\;and\;\Phi^{-}\left(a_{t}\right)>\Phi^{-}\left(a_{t^{\prime }}\right)\\ \Phi^{+}\left(a_{t}\right)<\Phi^{+}\left(a_{t^{\prime }}\right)\;and\;\Phi^{-}\left(a_{t}\right)<\Phi^{-}\left(a_{t^{\prime }}\right) \end{array}\right.$$

To resolve this issue, PROMETHEE II is utilized instead of PROMETHEE I, where PROMETHEE II provides a net outranking flow.

The net flow for each particular alternative ($\Phi^{net}\left(a_{t}\right))$ is calculated using Equation (8):

(8) $$\Phi^{net}\left(a_{t}\right)=\Phi^{+}\left(a_{t}\right)-\Phi^{-}\left(a_{t}\right)$$

PROMETHEE II can be used to obtain a net order determined by net flow, as presented below in statements (9) and (10).

(9) $$a_{t}\;\mathrm{is}\;\mathrm{preferred}\;\mathrm{to}\;a_{t^{\prime }}\;(a_{t}Pa_{t^{\prime }})\;\mathrm{if}\;\Phi^{net}\left(a_{t}\right)>\Phi^{net}\left(a_{t^{\prime }}\right)$$

(10) $$a_{t}\;\mathrm{is}\;\mathrm{indifferent}\;\mathrm{to}\;a_{t^{\prime }}\;(a_{t}Ia_{t^{\prime }})\;\mathrm{if}\;\Phi^{net}\left(a_{t}\right)=\Phi^{net}\left(a_{t^{\prime }}\right)$$

The greater the net $\Phi^{net}\left(a_{t}\right)$ value, the superior the alternative [28].

In our study, we utilized the F–PROMETHEE technique for the evaluation of tests for diagnosing UTIs. The selected test options were microscopy, nitrite, leucocyte esterase, catalase, culture 10^5^ bacteria/mL, chromogenic agar, biosensor, microfluidics, MALDI–TOF–MS, multiplex PCR, urine flow cytometry, sequence-based diagnostics, real-time microscopy systems, FISH, and immunologically based assay. The criteria used in the study were analytical, sensitivity, specificity, PPV, NPV, sample volume, type of urine (mid-stream or catheter specimens), test procedure (manual or automated) assay technique, detection techniques POC, and laboratory-based, cost, time to the first result, efficiency tests (low, moderate, or high), result acquisition (assessment by optic reader, assessment by direct observation, and outcome by system), interpretation of results (qualitative/quantitative/semi-quantitative), and AST, as shown in Table 1. The implementation performances were obtained from previous research in the literature for insertion into the F–PROMETHEE system for alternate assessment. The criteria and weights were defined according to the experts’ opinions with a triangular fuzzy linguistic scale. Extraordinary situations such as outbreaks were also taken into account in scoring and the importance levels of the test criteria were determined.

Triangular fuzzy numbers were defuzzied using the Yager index [34], which depends on the centre of weight, of the surface, and of the triangular membership function; the fuzzy numbers were compared and a suitable weight for each criterion was determined. Then, the Gaussian preference function was applied to prevent tiny deviations during the determination of the preference index of the alternatives, and the PROMETHEE analysis was performed.

## 3. Results

The $\Phi^{net}$ of each test was calculated by subtracting the negative outranking flow ($\Phi^{-}$) from the positive outranking flow ($\Phi^{+}$), where each alternative was numerically compared based on each criterion. Each alternative’s strength can be conceptualized numerically as the positive outranking flow, whereas the weak point of the alternatives can be thought of as the negative outranking flow. Therefore, the net flow produces the results of the net ranking; the higher the net flow, the more effective the alternative. The biosensor was the most indicative test expected for UTIs, with a net flow of 0.0063. The second-best option was the real-time microscopy system with a net flow of 0.003, whereas sequence-based diagnostics was the least effective alternative with a net flow of −0.0048 (given in Table 2) considering the selected parameters and weights. This ranking is an outcome of the primary supremacy of the following criteria: sensitivity, specificity, false positivity, false negativity, practicability, and efficiency. However, the outcomes could be different if every criterion was given a different weight or differentiated by the decision makers. Table 2 indicates the outcomes of the net ranking for the UTI diagnostic tests.

**Figure 3:**
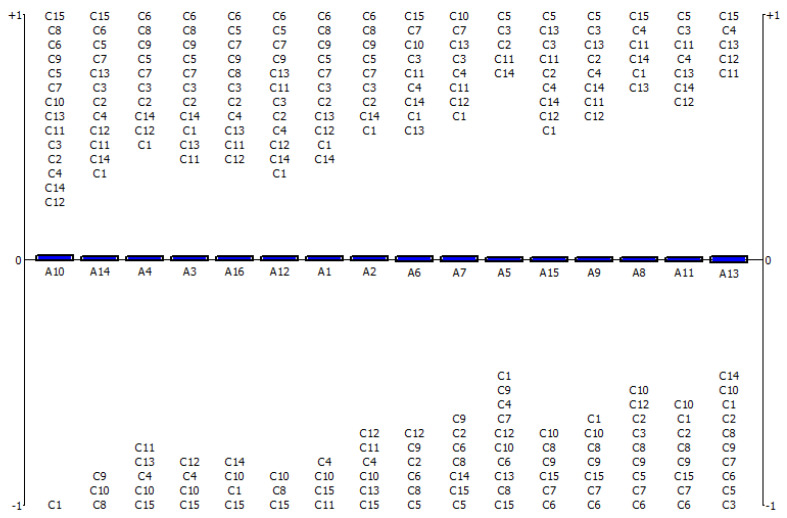
The evaluation of UTI diagnostic tests with F–PROMETHEE. **Abbreviations**: C1, specimen volume; C2, specimen type; C3, cost; C4, efficiency; C5, result time; C6, point of care testing; C7, applicability/direct from sample; C8, practicality for patients; C9, practicality for personnel; C10, limitation/pathogen identification; C11, sensitivity; C12, specificity; C13, PPV; C14, NPV; C15, antimicrobial susceptibility testing; A1, nitrite; A2, leucocyte esterase; A3, combined LE and nitrite; A4, catalase; A5, microscopy; A6, conventional culture; A7, chromogenic agar; A8, nucleic acid–based techniques (multiplex PCR); A9, MALDI–TOF–MS; A10, biosensor; A11, urine flow cytometry; A12, microfluidics; A13, sequence-based diagnostics; A14, real time microscopy systems; A15, FISH; A16, immunologically based assay.

Figure 3 shows a representation of each model and its relative effectiveness. The most preferred model is shown first, followed by the less preferred models. Therefore, the alternatives are ranked and presented from left to right. In Figure 3, the above-mentioned criteria represent the strengths of the alternatives, whereas the weaknesses of the alternatives are stated below the zero level. Thus, biosensor is the most preferred method, with almost all the criteria above the zero level, whereas sequence-based diagnostics is the last effective technique for diagnosing UTIs, with most of the criteria below the zero level.

For the sensitivity analysis, we omitted the criteria of specimen volume and specimen type and then regenerated the ranking results, as presented in Table 3.

## 4. Discussion

UTIs are among the most common infections acquired from a hospital. Clinical characteristics of UTIs are determined by the parts of the urinary tract affected, the etiological organisms, the extent of the infection, and the patient’s ability to deploy an immune response against it. UTIs, caused by many different microorganisms, can spread into the bloodstream, leading to bacteraemia and increased mortality [35]. Frequently used screening items such as dipstick testing for nitrite and LE in urine as well as microscopy analysis for bacteria and WBCs are fast but have low sensitivity. Therefore, rapid screening and accurate prediction are needed for immediate treatment. In the current study, we evaluated 16 different UTI test screenings with various criteria using MCDM theory to choose the most suitable and accurate techniques. We revealed that microfluidics should be the first diagnosis tool used to examine for UTI. According to the results of the study conducted by T. Wu et al., microfluidics was the primary screening test with high sensitivity for identifying UTIs [36]. W.P. et al. showed that microfluidics offers excellent diagnostic potential for UTIs with high sensitivity and fast turnaround time [37]. Additionally, as demonstrated in the study by A. O. Olanrewaju et al., microfluidic technology provided facile, rapid, and sensitive identification of bacteria in urine when compared with conventional methods [38]. In most diagnostic studies, it has been established that microfluidic devices have the capacity for the preliminary diagnosis of diseases and analysis of biological samples [23].

A recent study confirmed that the combination of microfluidics with FISH reduced reagent consumption and analysis time, as well as that FISH works well in microfluidic channels and is particularly effective for the diagnostics of fungal urinary tract infections [19]. In another study, it was shown that the micro-fluidic device allowed for quantitative determination and early detection of leukocyte esterase in human urine. The study showed that the concentrations of leukocyte esterase detected in urine were lower than concentrations detected with the ELISA leukocyte esterase kit [39]. Consistent with the literature, our study conducted with the MCDM technique revealed that microfluidic devices are a cheaper and more efficient alternative, as they only require the collection of small volumes of urine samples in the diagnosis of UTI. K. E. Mach et al. conducted a biosensor platform study based on the comparison between the biosensor test and traditional urine culture. The study presented the first clinical data that the POC test was successful in detecting infections in urine samples in a short time [40]. This was similar to our result, as biosensor was the second-best approach for diagnosing urinary tract pathogens. On the other hand, the study conducted by S. Reyes et al. revealed that microfluidic devices and FISH assays, which can save time in the detection of UTIs when compared with flow immunoassays and flow cytometry, were more favorable [8]. According to another study by MI, A. D., it was proven that the FISH method is simple, rapid, reliable, cheap, and able to specifically detect pathogens quantitatively [41]. This was consistent with our study, as the first and third-best options were microfluidic and FISH devices, respectively. Therefore, the performance of various systems must be constantly evaluated to determine the most accurate and appropriate techniques. Flow cytometers analyze urine by counting and sorting cells and can distinguish between red blood cells, white blood cells, bacteria, yeasts and epithelial cells. The new Sysmex UF-5000 fluorescence flow cytometry has the best capabilities to distinguish between Gram-positive and Gram-negative bacteria. There are many studies evaluating flow cytometers and urine culture in the diagnosis of UTIs. The new Sysmex UF-5000 fluorescence flow cytometry showed high diagnostic accuracy with a very low rate of false negatives, thereby reducing conventional culture workload and response time [19,29]. These studies are consistent with our study, as flow cytometry outperformed on conventional culture in terms of diagnostic accuracy and rapid response time.

MALDI–TOF–MS is a technique for pathogen identification from a culture that delivers accurate and rapid results. However, the main drawback is that initial reagent and instrument costs are high, limiting the use of this platform to high-volume laboratories. Additionally, it requires a pure culture of bacteria and uses isolated bacterial colonies following standard urine culture [4,39]. PCR analysis is used to extract the genetic material from a positive clinical sample and can detect many bacterial pathogens and species of yeast. Although PCR delivers significant time savings over conventional urine culture, this technique is not suitable for the detection of UTI pathogens, as it is specifically designed for the detection of pathogens such as sexually transmitted pathogens. The current PCR assays only provide qualitative data, thus indicating only the presence of bacteria and not bacterial concentration. However, the quantification of bacteria in a urine specimen is necessary to distinguish between contamination and infection and to guide clinical decision making [35].

In our study, the last three techniques—microscopy, leukocyte esterase, and nitrite− were the least effective for diagnosing UTIs. The main defect of these tests is their poor predictive values. In some cases, some Gram-positive pathogens do not produce nitrites, such as *Staphylococcus* spp. and *Enterococcus* spp.; elevated urinary white blood cells (WBCs) may result due to contamination of the female genital tract; patients with low immunity may have normal urinary WBC levels; and bacteria may be present due to contamination with perianal, vaginal, epidermal, and periurethral specimens. For this reason, these tests are not used as POC tests but are used together with other tests in the clinical laboratory.

The MCDM methods have been extensively used in various fields to remove mystery in the choice of appropriate alternatives for diagnosis [22,26,32]. F–PROMETHEE is one of the most recently developed and successful approaches for ranking options under a fuzzy environment [26,41]. Considering the vagueness in the medical and health fields, this approach is very beneficial for both experts and patients [32]. It is a supportive system that systematically analyses the differences between the options and provides decision points [32]. In the MCDM process, assigning weights to the criteria is not an easy task. In this study, a fuzzy linguistic scale was used to specify the weights based on experts’ experience and opinions. Additionally, the same linguistic scale was used to simplify the criterion, which contains vague data. According to the significance of each criterion (not every criterion is equally important), it was necessary to assign weights to assess alternatives during the decision-making process. Therefore, the most crucial criteria were assigned greater weight, while the least important criteria were given less weight [32]. To the best of our knowledge, this is the first study to suggest in the literature that the MCDM (FPROMETHEE) technique could be applied to assess the performance and efficiency of commercially available UTI tests. This enables decision makers to assess multiple criteria simultaneously in various UTI tests and decide on the preferred instruments or techniques to decrease the risk of infection, increase rates of cure, and prevent deaths. In terms of the limitations of our study, the ranking was determined according to the selected alternatives, criteria, and the weights given to these criteria. Selected data and results can be reconstructed and updated according to the decision maker’s needs or circumstances. This study demonstrated the MCDM process’s applicability in evaluating UTI tests and also highlighted their positive and negative features.

## 5. Conclusions

This study used fuzzy-based MCDM approaches to analyze the sixteen available UTI tests based on the chosen criteria. The assessments of the alternatives’ efficacy were made, and their associated weights were decided by experts. According to the assessment findings, the biosensor was the most effective diagnostic technique. The top three UTI diagnostic tests were determined to be biosensors, real-time microscopy systems, and catalase, whereas the last efficient diagnostic technique was sequence-based diagnostics. During the assessment of the effectiveness of the available UTI diagnostic tests, every technique was considered based on its general characteristics combined with the most recently published guidelines. The results demonstrated the effectiveness of the new techniques in diagnosing UTIs. The F–PROMETHEE method can aid decision makers in making decisions on the most suitable UTI tests to support the outcomes of each country. This method will also help guide manufacturing companies and medical laboratories in producing and using the best alternatives for the future.

## Figures and Tables

**Table 1:** Criteria of the UTI test diagnosis options and their significance weights with linguistic triangular fuzzy scale.

Linguistic Scale for Evaluation	Triangular Fuzzy Scale	Criteria
Very High	(0.75, 1, 1)	Result time (C5), point of care testing (C6)
High	(0.50, 0.75, 1)	Cost (C3), efficiency (C4), limitation/pathogen identification (C10), sensitivity (C11), specificity (C12), PPV (C13), NPV (C14), antimicrobial susceptibility testing (C15)
Moderate	(0.25, 0.50, 0.75)	Applicability/direct from samples (C7), practicality for patients (C8), practicality for personnel (C9)
Low	(0, 0.25, 0.50)	Specimen type (C2)
Very Low	(0, 0, 0.25)	Specimen volume (C1)

Abbreviations: PPV: positive predictive value; NPV: negative predictive value.

**Table 2:** Complete ranking outcomes of UTI diagnostic tests with F–PROMETHEE.

Rank	UTI Tests	$\Phi^{net}$	$\Phi^{+}$	$\Phi^{-}$
1	Biosensor	0.0063	0.0064	0.0001
2	Real-time microscopy systems	0.003	0.0041	0.0011
3	Catalase	0.0026	0.004	0.0014
4	Combined leucocyte esterase and nitrite	0.0025	0.0039	0.0014
5	Immunologically based assay	0.002	0.0031	0.0011
6	Microfluidics	0.0019	0.0031	0.0012
7	Nitrite	0.0018	0.0039	0.0021
8	Leucocyte esterase	0.0018	0.0039	0.0021
9	Conventional Culture	0.0004	0.0038	0.0034
10	Chromogenic agar	−0.0021	0.0025	0.0045
11	Microscopy	−0.0022	0.0013	0.0035
12	FISH	−0.0029	0.0012	0.0041
13	MALDI–TOF–MS	−0.003	0.0011	0.0042
14	Nucleic acid-based techniques (Multiplex PCR)	−0.0036	0.0013	0.0049
15	Urine Flow cytometry	−0.0037	0.0008	0.0045
16	Sequence-based diagnostics	−0.0048	0.0029	0.0077

Abbreviations: UTI, urinary tract infection; FISH, fluorescence in situ hybridization; MALDI–TOF–MS, matrix-assisted laser desorption ionization-time of flight; PCR, polymerase chain reaction.

**Table 3:** Sensitivity analysis ranking outcomes of UTI diagnostic tests with F–PROMETHEE.

Rank	UTI Tests	$\Phi^{net}$	$\Phi^{+}$	$\Phi^{-}$
1	Microfluidics	0.2182	0.2194	0.0012
2	Biosensor	0.1925	0.1998	0.0073
3	FISH (Flourescent in situ hybridisation)	0.1337	0.1658	0.0321
4	Real-time microscopy systems	0.0808	0.1595	0.0787
5	Urine Flow cytometry	0.0651	0.1419	0.0768
6	Chromogenic agar	0.0569	0.1565	0.0997
7	MALDI–TOF–MS	0.0364	0.1269	0.0905
8	Nucleic acid-based techniques (Multiplex PCR)	−0.0087	0.1091	0.1178
9	Conventional Culture	−0.0241	0.0992	0.1233
10	Catalase	−0.0282	0.1032	0.1314
11	Sequence-based diagnostics	−0.0311	0.1067	0.1378
12	Immunologically based assay	−0.1112	0.0733	0.1845
13	Nitrite	−0.1212	0.0702	0.1914
14	Combined leucocyte esterase and nitrite	−0.1335	0.0563	0.1899
15	Microscopy	−0.1513	0.058	0.2093
16	Leucocyte esterase	−0.1743	0.0439	0.2182

The ranking results have not changed after the sensitivity analysis, indicating that the initial ranking was robust and not significantly influenced by variations in input parameters.

## Data Availability

The datasets generated and analysed during the current study are available from the corresponding author on reasonable request.
